# The relationship between perceived restorativeness and place attachment for hikers at Jeju Gotjawal Provincial Park in South Korea: the moderating effect of environmental sensitivity

**DOI:** 10.3389/fpsyg.2023.1201112

**Published:** 2023-08-07

**Authors:** Jee In Yoon, Soyoun Lim, Mi-Lyang Kim, Jinyoung Joo

**Affiliations:** ^1^Department of Coaching, College of Physical Education, Kyung Hee University, Yongin, South Korea; ^2^Department of Kinesiology, Mississippi State University, Mississippi State, MS, United States; ^3^Department of Sport, Leisure and Recreation, Soonchunhyang University, Asan, South Korea; ^4^Center for Happiness Studies, Seoul National University, Seoul, South Korea

**Keywords:** perceived restorativeness, place attachment, attention restoration theory, outdoor recreation, Gotjawal

## Abstract

**Objectives:**

Jeju Gotjawal Provincial Park provides visitors with opportunities for outdoor recreation and informs visitors of the environmental significance of the park’s ecosystem. This study attempted to examine how the perceived restorativeness of park visitors influenced their place attachment. In addition, the moderating effect of environmental sensitivity on the hypothesized relationship was explored.

**Methods:**

Using the purposive sampling method, 408 surveys were collected at Jeju Gotjawal Provincial Park. The hypotheses were tested by confirmatory factor analysis, path analysis, and invariance tests using Lisrel 8.70.

**Results:**

The results indicated that perceived restorativeness had a positive influence on place attachment (place identity and place dependence). Further, the hypothesized relationship was stronger for the visitors with higher environmental sensitivity, compared to those with weaker environmental sensitivity.

**Conclusion:**

Park managers should consider ways to increase the perceived restorativeness of visitors as they experience the natural environment at the park. Also, since environmental sensitivity played an important role in shaping the perceived restorativeness–place attachment relationship, there is a need for educational programs that can inform visitors of the significance of the natural environment to increase their affection for nature.

## 1. Introduction

A lack of green space in urban areas can result in nervousness, lethargy, and depression in urban residents (Olmstead, 1865). Nonetheless, urbanization and the subsequent loss of green space in downtown areas continue to increase (Lin et al., 2015). As a result, the per capita green area ratio has inevitably decreased because of overcrowding in urban areas (Haaland and van Den Bosch, 2015; Lin et al., 2015). Due to the overcrowding, urban residents often face psychological problems (e.g., depression, mental illness) (Berry, 2007). South Korea, where more than 90% of the population lives in metropolitan areas (Ministry of Land, 2020), is no exception. It has been continuously reported that problems caused by a lack of green space can negatively affect individuals’ psychological (Wolch et al., 2014; Spano et al., 2021) and physical health (Richardson et al., 2013; Kondo et al., 2018).

According to recent studies, when individuals enjoy leisure activities in a natural environment, they can not only relieve stress and reduce depression (Siefken et al., 2019; Shobri et al., 2021; Quarta et al., 2022), but also gain psychological benefits (Buchecker and Degenhardt, 2015; Marselle et al., 2019) and maintain physical health (Kondo et al., 2018; Winter et al., 2019). The process through which an individual restores their physical and mental well-being while spending time in the natural environment is called “perceived restorativeness.” According to the attention restoration theory (ART), individuals can feel their attention restored while interacting with the natural environment. This process increases psychological resilience, which plays a very important role in controlling and reducing stress, fatigue, and anxiety (Buchecker and Degenhardt, 2015; Frumkin et al., 2017; Marselle et al., 2019). Perceived restorativeness, the central concept of ART, means that individuals can overcome mental fatigue and lower psychological stress by themselves without making separate conscious efforts (Ohly et al., 2016). Other researchers have consistently reported that spending time in a natural environment benefits people’s health (Buckley et al., 2019; Li et al., 2021; Buckley and Cooper, 2022).

Participating in outdoor recreational activities provides a good opportunity to enjoy physical activities while also having a positive psychological experience in nature (Brown, 2019). Researchers have been interested in positive psychological states such as perceived restorativeness that can be developed through interaction with nature (Andre et al., 2017). A previous study (Stack and Shultis, 2013) showed that park visitors in urban areas could reduce their stress and gain psychological benefits in natural spaces. Researchers (Weng and Chiang, 2014) also found that outdoor leisure activities can reduce anxiety from daily life and thereby help to improve physical and mental health. They identified an association between the function of perceived restorativeness and the reduction of anxiety. Moreover, nature-based recreation activities were found to play a better role than indoor leisure activities in calling attention to and feeling perceived restorativeness. When individuals are placed in a natural environment, their attention turns to the natural landscape, which reduces negative thoughts (Ulrich et al., 1991). Furthermore, in natural outdoor spaces, individuals can replace their negative emotions with positive ones while restoring balance in their bodies (Hansmann et al., 2007).

As people repeatedly encounter nature, they may develop emotional attachments or bonds to a place, which is referred to as place attachment. It is mainly comprised of two dimensions: one’s emotional and symbolic attachment to a place (place identity) and the functional attachment to a setting (place dependence). It is well known that positive psychological experiences formed through interactions with nature can contribute to an increase in either form of place attachment (Vada et al., 2019; Yan and Halpenny, 2022). One of these positive psychological experiences is perceived restorativeness, the core concept of ART. Outdoor recreationists can experience perceived restorativeness in the places they repeatedly visit for a certain period, which can contribute to developing place attachment (Beery and Jönsson, 2017). Thus, the more restorative the natural environments available for outdoor recreationists, the more their place attachment can be developed (Stack and Shultis, 2013). Thus, the perceived restorativeness experienced in nature is positively associated with place attachment (Korpela and Hartig, 1996).

In South Korea, Jeju Island is considered a tourism and outdoor recreation center because of its unique ecosystem and spectacular natural scenery. Due to its variety of natural environments, such as the sea, mountains, and waterfalls, Jeju Gotjawal Provincial Park represents a unique ecosystem that may only be found in Jeju. The term “gotjawal” is a word in the native language of the Jeju province that generally refers to areas where forests were formed on top of rocky areas created by volcanic activity. Because these areas are environmentally sensitive and unique, Jeju has autonomously designated some of these “gotjawal” areas as parks to protect them. Although “gotjawal” holds particular significance to visitors, few researchers have paid attention to these settings and their influence on visitors. Thus, for the optimal management of natural resources, researchers need to pay attention to further understanding the perceived restorativeness experienced by visitors to Jeju Gotjawal Provincial Park and how this perceived restorativeness increases place attachment. Therefore, the present study aims to examine how the perceived restorativeness of visitors to Jeju Gotjawal Provincial Park affects their place attachment.

A nature-based recreation setting is always accompanied by a dual mandate—preserving natural resources in the relevant areas while ensuring the pleasure of leisure participants (Pouwels et al., 2017). This means that considering how to preserve natural places for leisure activities is essential to sustaining their benefits to visitors. Encouraging visitors with preservation activities can effectively aid in preserving natural places. According to previous studies, to promote the preservation activities of visitors, inspiring positive environmental attitudes of visitors toward places of outdoor leisure activities is crucial (Lee and Jan, 2015; DeVille et al., 2021). This is because the positive environmental attitudes of visitors may naturally be linked to long-term efforts of preservation activity (Rosa and Collado, 2019; DeVille et al., 2021). Among various positive environmental attitudes, environmental sensitivity refers to the degree of positive and friendly feelings toward natural environments. Environmental sensitivity is closely related to people’s environmental concerns (Metzger and Mcewen, 1999). According to previous studies, people with higher environmental sensitivity are more interested in environmental issues and more likely to participate in efforts to preserve natural environments than people with lower sensitivity (Bodur and Sarigöllü, 2005; Yilmaz et al., 2009). Therefore, examining how environmental sensitivity influences the relationship between perceived restorativeness and place attachment can provide an in-depth understanding of visitors and their preservation behaviors in a nature-based recreation setting.

**Figure 1:**
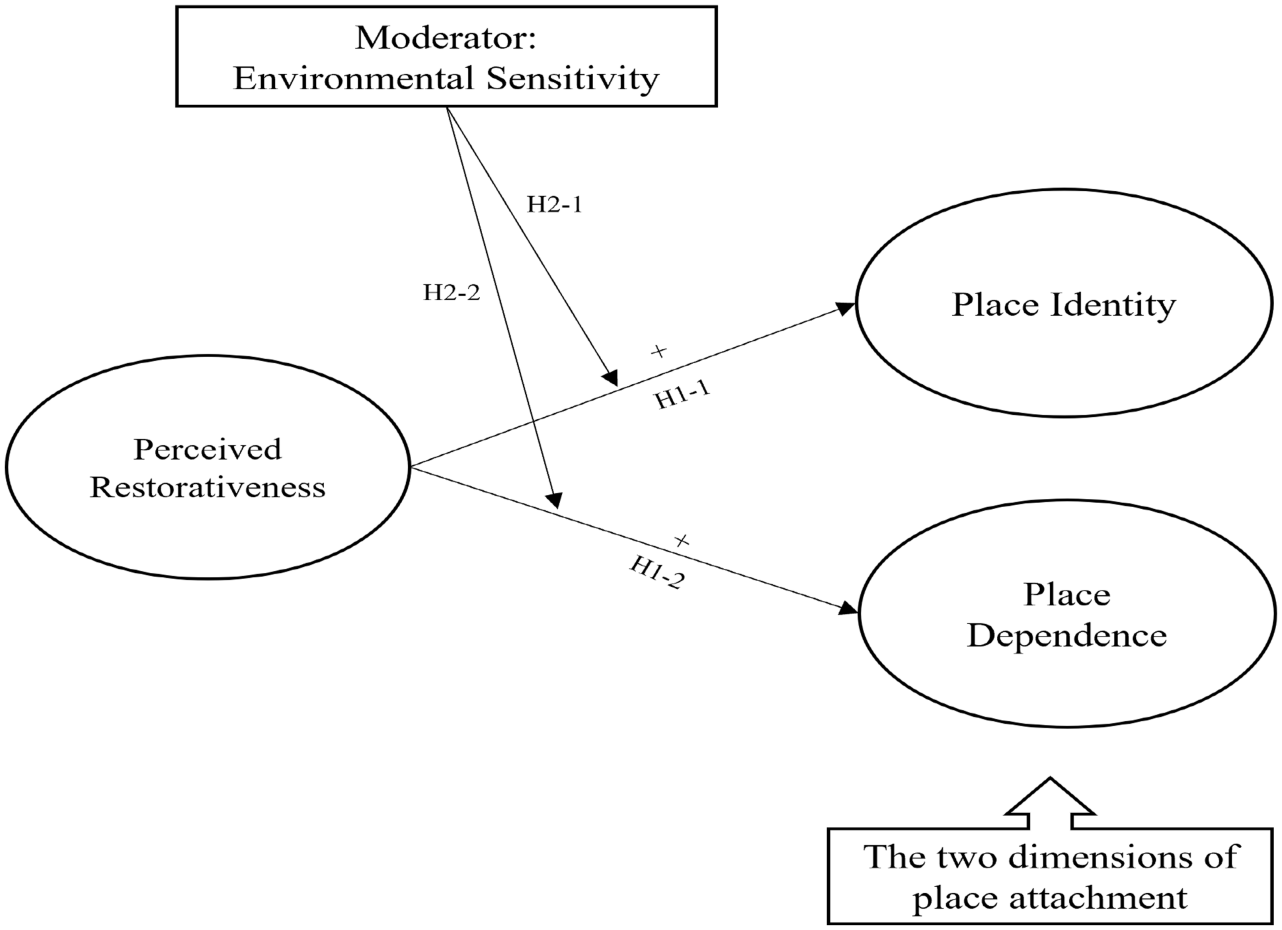
Hypothesized model.

The current study investigated (1) how the perceived restorativeness of visitors to Jeju Gotjawal Provincial Park positively affects their place attachment, and (2) how the relationship between perceived restorativeness and place attachment varies due to the level of environmental sensitivity. According to previous studies, the higher the environmental awareness, such as environmental sensitivity, the higher the sense of recovery and place attachment felt in outdoor leisure activities. Accordingly, this study assumed that, for the group with high environmental sensitivity, their perceived restorativeness would have a stronger impact on place attachment than those with low environmental sensitivity. For testing the second hypothesis (moderating effect of environmental sensitivity), visitors to Jeju Gotjawal Provincial Park were divided into two groups of high and low environmental sensitivity. Then, testing was conducted to examine how the relationship between perceived restorativeness, and place attachment differed between the two groups (see Figure 1).

*Hypothesis 1. Perceived restorativeness will positively influence place attachment.*

*H1-1. Perceived restorativeness will positively influence place identity.*

*H1-2. Perceived restorativeness will positively influence place dependence.*

*Hypothesis 2. Environmental sensitivity will have a moderating effect on the relationship between perceived restorativeness and the two dimensions of place attachment (place identity and place dependence).*

*H2-1. The perceived restorativeness will have a stronger positive influence on place identity for the high environmental sensitivity group than the low environmental sensitivity group.*

*H1-2. The perceived restorativeness will have a stronger positive influence on place dependence for the high environmental sensitivity group than the low environmental sensitivity group.*

## 2. Literature review

### 2.1. Perceived restorativeness

ART assumes humans can feel positive experiences and psychological recovery by spending time in nature (Basu et al., 2019). The core concept of ART is “perceived restorativeness,” which is a physical, mental, and social recovery process in which a negative state of mind can be recharged and restored through interaction with nature. To be a restorative environment, it must be accessible and away from everyday life, and that new environment has a large enough scale to provide a sense of recovery without requiring directed attention (Kwon et al., 2017). The natural environment is a representative restorative environment. Green landscapes, such as forests, mountains, streams, and oceans, can be viewed with involuntary attention that requires no specific effort. Therefore, looking at and feeling the natural environment removes daily stress and physical, mental, and emotional fatigue and positively affects psychological health and attention recovery (Yi, 2023).

There are four aspects of perceived restorativeness. First, “being away” is physically or psychologically distancing oneself from daily activities that may cause attention fatigue (Felsten, 2009). Second, “fascination” is an environment attractive enough to unintentionally draw an individual’s attention without specific efforts. It refers to stimulation that provides aesthetic pleasure coming from the natural environment. Third, “compatibility” is a recovery that can occur when one’s needs correspond to what the environment provides (Pasanen et al., 2018). It occurs when the leisure setting fits what one is trying to achieve (e.g., recreational goals meet demands in the setting). “Extent” means an environment that can be felt in nature, rich enough to be perceived as “the whole other world” and enough to see and think about to occupy one’s mind completely. This study used the perceived restorativeness scale (PRS) (Hartig, 1996) to measure these four aspects of the perceived restorativeness of the natural environment and has been verified by many studies (Pasini et al., 2009; Peschardt and Stigsdotter, 2013; Negrín et al., 2017).

### 2.2. Place attachment

Place attachments, first described in detail in a 1992 edited volume by Altman and Low, involve affective bonds to places across multiple geographic scales, with a variety of temporal qualities and social actors and processes contributing to the bonds (Brown et al., 2012). The conceptualization of place attachment is diverse, generating multiple theoretical structures and relationships with other concepts, such as environmental behaviors (Hidalgo and Hernandez, 2001; Hernández et al., 2020). Specifically, place attachment refers to the psychological bonding between an individual and a meaningful environment (van Riper et al., 2019; Hernández, 2021). Although place attachment is a psychological bond between people and place, it also includes individuals’ thoughts about the characteristics of physical space. This idea is essential to what “experience” you have had (Scannell and Gifford, 2010a,b). Experience accumulates during repeated visits to one place, and based on such experiences, the physical space can be imprinted on the individual as a meaningful place. Early studies on attachment to place mainly dealt with residents’ attachment to the local community (Hummon, 1992; Williams et al., 1995). However, studies on visitors’ attachment to places related to leisure or outdoor recreation are also being actively conducted (Budruk and Stanis, 2013; Hernández, 2021). In addition, Lewicka (2011) pointed out that the studies on place attachment in recent decades have been conducted, especially in nature-based areas such as forests (Smaldone, 2006), lakes (Jorgensen and Stedman, 2006), seacoasts (Kelly and Hosking, 2008), mountains (Kyle G. et al., 2003; Kyle G. T. et al., 2003), and wilderness settings (Wynveen et al., 2012). A study has shown that the greener spaces are included in the surrounding environment, the more critical it is to place attachments (Weber et al., 2008).

Scannell and Gifford (2010a,b) conceptualized place attachment from a natural resource management perspective, emphasizing people’s interpretation and interaction with nature. They noted that neglecting the emotional and symbolic values humans ascribe to the environment will negatively influence the experience of nature (Williams et al., 1992). In previous studies (Williams et al., 1992; Proshansky et al., 2014), place attachment is the most representative concept. It has two dimensions. The first dimension is place identity, which means that the self is satisfied at the center of why the place becomes vital to the individual. In other words, whether the place can help one realize self-identity while doing leisure activities in a place is an important criterion. In addition, the individual’s feelings and emotions in the place play an important role when building place identity. It is an emotional attachment to a landscape. A previous study (Schreyer et al., 1981) explained an emotional-symbolic dimension of place attachment (place identity) because individuals perceived the place as a central aspect of subjects’ lives (Kyle G. et al., 2003; Kyle G. T. et al., 2003).

The second dimension of place attachment is place dependence. It refers to the attachment to landscapes from the perspective that some settings support individual needs better than others (van Riper et al., 2019). In other words, place dependence is a functional attachment to the place. The items measuring place dependence indicate whether respondents were less willing to visit another site for their leisure activity. A previous study (Bricker, 1998) found that recreationists with higher place dependence were more likely to support management options such as developing amenities and trails. However, the study findings indicate that recreationists with higher place identities were reluctant to support those management changes. Previous studies have revealed various psychological benefits individuals can obtain by having an attachment to a place (Scannell and Gifford, 2017). For example, outdoor places provide opportunities to relax in nature (e.g., Fishwick and Vining, 1992) and functional conveniences that enable various activities. This functional convenience was named place dependence in the study of attachment to place (e.g., Kyle et al., 2005), and it can be further strengthened when the physical and social convenience provided by the place matches well with the purpose pursued by the visitor.

Previously, these two dimensions of place attachment (i.e., place identity and place dependence) were recognized to be the most reliable across various samples (Moore and Graefe, 1994; Warzecha and Lime, 2000). Both dimensions of place attachment refer to a sense of psychological belonging between humans and places. However, it should be noted that the content and role of these two measures differ: place identity is emotional, and place dependence is functional.

### 2.3. Moderator: environmental sensitivity

Environmental sensitivity is a set of affective attributes that may result in an individual perceiving environmental concern empathetically (Perterson, 1982). Environmental sensitivity is a construct that affects place attachments and environmental attitudes toward environmentally friendly behavior (Saputra and Putra, 2020). Even though it promotes environmental stewardship behaviors, environmental sensitivity has received relatively less attention from researchers (Ananda and Hampf, 2015). Sia et al. (1986) insisted that environmental sensitivity and skills/knowledge about environmental behaviors were the significant antecedents of pro-environmental behaviors. Further, more attention is necessary to explore environmental sensitivity to increase pro-environmental behaviors (Metzger and Mcewen, 1999). A previous study (Saputra and Putra, 2020) showed that environmental sensitivity had a significant and positive effect on place attachment. Further, environmental sensitivity and place attachment both positively influenced environmentally responsible behavior. If tourists are highly sensitive to the environment, they are more likely to show an environmentally responsible attitude (Chawla, 1998). Similarly, tourists’ environmental sensitivity had a significant positive effect on place attachment (Cheng and Wu, 2015). Therefore, it is likely that one with higher environmental sensitivity will show higher place attachment. Previous studies lacked interest in environmental sensitivity and its influence on place-related constructs of outdoor recreation participants. Studies suggested the possible moderators of place attachment-environmental behavior relationship. That is, factors that can change place-attached individuals or groups into pro- or anti-environmental actors (Carrus et al., 2014). Carrus et al. (2014) also noted that one of the possible moderators is a positive attitude toward nature or a concern about environmental problems. In addition, Vaske and Kobrin (2001) said that environmental citizenship, which can be positively influenced by environmental sensitivity, is essential in predicting eco-friendly behavior or place attachment. Therefore, this study aims to verify whether the environmental sensitivity of hiking participants in Jeju Gotjawal Provincial Park affected the intensity of the place attachment.

## 3. Materials and methods

### 3.1. Data collection

Data collection was performed at Jeju Gotjawal Provincial Park in October 2021. Korea has four distinct seasons, and Jeju Island’s average temperature in October is 15.5 degrees Celsius (59.9 in Fahrenheit), the most pleasant autumn weather. We selected October as the data collection period based on October’s peak number of visitors to the park and the pleasant weather conditions. No statistical data were officially compiled or provided by Jeju Gotjawal Provincial Park. However, the park management office recommended the month with the most visitors is October (approximately 25,000 in October 2021). In addition, Jeju Island had the highest number of tourists in October, and the number of Jeju tourists in October reached 1.2 million (Jeju tourism association, 2021). “Gotjawal” are forests created on irregular rocks created by lava flows that erupted during periods of volcanic activity. These forests have a variety of flora and fauna only found in Jeju. Gotjawal Provincial Park is one of the four major “gotjawal” areas and is in the inland area of southwestern Jeju. The park is in Daejeong-eup, Seogwipo-si, and covers approximately 1.54 million square meters. Purposive sampling was used to collect data from visitors hiking at the park. The park offers a total of five hiking courses. Four research assistants were located at the entrance/exit point of the hiking trails. Since there is only one entrance/exit point vin the park, the assistants were located there to get consent from the survey participants. After obtaining agreement for participating in the on-site survey, assistants provided questionnaires to study participants. Before participants answered the survey, assistants explained the purpose of the study. The data collection was conducted from 9 a.m., the park’s opening time, and completed before 2 p.m. The peak usage time for hiking participants, advised by park managers, is between 10 a.m. and 1 p.m. A total of 408 copies of the questionnaire were completed by respondents and used in the final analysis.

### 3.2. Measures

Perceived restorativeness was originally measured by PRS (Hartig et al., 1997). In the Korean context, Yi (2023) used the revised version of PRS containing diverse dimensions of perceived restorativeness. Based on Yi (2023) revised version of PRS, this study used four survey items to measure the four dimensions of perceived restorativeness (being away, fascination, extent, and compatibility). These items captured the positive psychological feelings of restoration perceived from the park (e.g., “Jeju Gotjawal Provincial Park is a place where I can get out of my daily life and relax and think about my favorite things”). As shown in Table 1, RST1 measured being away, RST2 for fascination, RST3 for extent, and RST4 for compatibility. Place attachment was measured using items adapted from the literature (Kyle et al., 2004, 2005) and included dimensions of place identity (four survey items) and place dependence (four survey items). The items for place identity measured the visitors’ identity as perceived in the park while place dependence assessed their functional attachment to the site. Place identity assessed recreationists’ emotional attachment to the park (e.g., “I have a strong sense of belonging in regard to Jeju Gotjawal Provincial Park”). On the other hand, place dependence items examined their functional attachment to the setting (e.g., “I am more satisfied with visiting Jeju Gotjawal Provincial Park than other hiking destinations”). Environmental sensitivity (four survey items) was measured by the visitors’ attitude toward the park’s natural environment and was based on a scale used in a previous study (Sivek, 2002). These items consisted of 5 items examining general positive attitudes toward the park’s natural environment (e.g., “I appreciate the natural environment of Jeju Gotjawal Provincial Park”). All items were rated on a 5-point Likert-type scale (1 = strongly disagree to 5 = strongly agree). Additionally, the survey included questions about respondents’ sociodemographic information such as age, gender, income, and education level.

**Table 1:** Means, standard deviations, internal consistencies, and factor loadings for perceived restorativeness, place identity, and place dependence.

Variables/Survey items	*SNF*				*ɑ*	*M*	*SD*	*λ*	*SE*
Perceived restorativeness				
RST1. Jeju Gotjawal Provincial Park is a place where I can get out of my daily life and relax and think about my favorite things.	4.44	0.695	0.659	0.032	0.843
RST2. Jeju Gotjawal Provincial Park is a place where everything is well organized without being crowded or disorderly.	4.24	0.787	0.767	0.035
RST3. Jeju Gotjawal Provincial Park is a sufficiently wide and free space without restrictions on movement.	4.03	0.949	0.817	0.041
RST4. In Jeju Gotjawal Provincial Park, it is convenient to find a way or move around, and I can do what I like.	4.11	0.820	0.796	0.036
Place identity				
PI1. Visiting Jeju Gotjawal Provincial Park has a deep meaning for me.	3.76	0.970	0.832	0.034	0.946
PI2. I have a strong sense of identifying with Jeju Gotjawal Provincial Park.	3.29	1.119	0.903	0.038
PI3. I have a strong sense of belonging in regard to Jeju Gotjawal Provincial Park.	3.42	1.114	0.916	0.037
PI4. I have a special feeling for Jeju Gotjawal Provincial Park.	3.52	1.127	0.935	0.037
Place dependence				
PD1. I enjoy traveling in Jeju Gotjawal Provincial Park more than other hiking destinations.	3.59	1.039	0.886	0.036	0.940
PD2. I am more satisfied with visiting Jeju Gotjawal Provincial Park than other hiking destinations.	3.63	0.978	0.881	0.034
PD3. It is more important to visit Jeju Gotjawal Provincial Park than other hiking destinations.	3.56	1.034	0.842	0.035
PD4. No other locations can replace the hiking of Jeju Gotjawal Provincial Park.	3.27	1.127	0.845	0.040
Environmental sensitivity				
ES1. I enjoy the natural environments of Jeju Gotjawal Provincial Park.	4.58	0.626	–	–	–
ES2. I am interested in the ecological preservation in Jeju Gotjawal Provincial Park.	4.22	0.800	–	–
ES3. I appreciate the natural environment of Jeju Gotjawal Provincial Park.	4.16	0.913	–	–
ES4. I care about the impact of my living habits on the natural environments of Jeju Gotjawal Provincial Park.	3.84	1.028	–	–

Scales ranged from 1 (Strongly Disagree) to 5 (Strongly Agree).

Fit statistics: *χ*^2^ = 111.361, df = 49, RMSEA = 0.057, NNFI = 0.988, CFI = 0.993.

### 3.3. Correlation between variables

Table 2 shows the results of the Pearson correlation analysis. There were all positive correlations between perceived restorativeness, place identity, place dependence*, and environmental sensitivity*. In addition, the correlation coefficient between each variable was between 0.570–0.717 (*p* < 0.01); there was no problem with the construct validity (Kline, 2013).

**Table 2:** The results of correlation analysis.

	1	2	3	4
Perceived restorativeness (1)	1			
Place identity (2)	0.570^**^	1		
Place dependence (3)	0.535^**^	0.717^**^	1	
Environmental Sensitivity (4)	0.637^**^	0.634^**^	0.571^**^	1

^**^p < 0.01.

### 3.4. Analysis

We used confirmatory factor analysis (CFA) to validate the theorized factor structure of our measurement model, and the goodness-of-fit index was used to confirm the variable composition. The measurement model was assessed using the following goodness-of-fit indices: root mean square error of approximation (RMSEA under 0.10) (Mac Callum et al., 1996), non-normed fit indices (NNFI greater than 0.90) (Hu and Bentler, 1998), and comparative fit indices (CFI greater than 0.95) (Hu and Bentler, 1998). All analyses were conducted using LISREL 8.70. Based on the CFA, all three variables (two dimensions for place attachment, and perceived restorativeness) showed a good fit (*χ^2^* = 111.361, df = 49, RMSEA = 0.057, NNFI = 0.998, CFI = 0.993) with the 12 survey items. All indices of the survey data met the fit criteria, confirming that each factor explained the measurement model. All Cronbach’s alpha coefficients were greater than 0.80 (Hair et al., 1998), which confirmed the internal consistency of the measured variables (see Table 1). In addition to the hypothesized model testing (relationship between perceived restorative ness and two place attachment dimensions), we also tested whether environmental sensitivity, one type of environmental attitude moderate, the relationship. Previous researchers (Bodur and Sarigöllü, 2005; Yilmaz et al., 2009) noticed that people with different levels of environmental sensitivity showed different degrees of perceived restorativeness in a natural setting and attachment to natural places. To test this moderation effect, we divided the pooled sample into the high environmental sensitivity group and the low environmental sensitivity group. We used a median score of environmental sensitivity to divide the group. Specific steps for the invariance testing (moderating effect of environmental sensitivity) are suggested in the results.

## 4. Results

### 4.1. Descriptive analyses

As shown in Table 3, a total of 64.8% of respondents were women. The respondents’ ages ranged from 10 to 79, and the mean age was 46.54 (*SD* = 13.64). About half of the respondents were university graduates (51.8%). 25.8% of respondents had an office job and 21.9% of respondents were professionals. About 21% of respondents’ annual average income was more than 70,000,000 KRW (approximately USD 65,800) (Kim et al., 2015). The mean and standard deviation of all items of the variables included in the research model are presented in Table 1.

**Table 3:** Socio-demographic characteristics.

		Valid percent
Gender	Male	35.2%
	Female	64.8%
Birth year	1940s	1.3%
	1950s	13.2%
	1960s	26.4%
	1970s	22.8%
	1980s	16.8%
	1990s	18.3%
	2000s	1.3%
Education	Elementary—high school	17.3%
	Two-year college	10.2%
	University	51.8%
	Master’s degree and more	20.8%
Occupation	Student	5.6%
	Corporate employee	25.8%
	Self-employment	10.5%
	Professional	21.9%
	Freelance	6.9%
	Preparing for employment	0.3%
	Other	14.5%
	Unemployed	14.5%
Annual income	<KRW 10,000,000 (~USD 9,400)	9.8%
	KRW 10,000,000–19,990,000	4.6%
	KRW 20,000,000–29,990,000	11.7%
	KRW 30,000,000–39,990,000	15.8%
	KRW 40,000,000–49,990,000	15.5%
	KRW 50,000,000–59,990,000	12.5%
	KRW 60,000,000–69,990,000	9.0%
	>KRW 70,000,000	21.0%

### 4.2. Model testing for pooled sample (H1 and H2)

For the pooled sample (*n* = 408), both the measurement model (χ^2^ = 264.767, df = 51, RMSEA = 0.084, NNFI = 0.971, CFI = 0.977, SRMR = 0.078) and path model (χ^2^ = 111.361, df = 49, RMSEA = 0.057, NNFI = 0.991, CFI = 0.993, SRMR = 0.032) showed an acceptable fit for the data (see Table 4). Hypotheses 1 and 2 were supported by the analyses (see Table 5). First, the perceived restorativeness of visitors at Jeju Gotjawal Provincial Park positively influenced place identity (H1; *β* = 0.618, *p* < 0.001) and 38.2% of the variance in place identity was explained by perceived restorativeness. Second, visitors’ perceived restorativeness had a positive effect on place dependence (H2; *β* = 0.567, *p* < 0.001). The explained variance was 32.1% for place dependence.

**Table 4:** Fit indices for the baseline models.

Model		*χ* ^2^	df	RMSEA	NNFI	CFI	SRMR
Pooled (*n* = 408)	Measurement model	264.767	51	0.084	0.971	0.977	0.078
Pooled (*n* = 408)	Path model	111.361	49	0.057	0.991	0.993	0.032
High environmental sensitivity group (*n* = 225)		126.967	49	0.061	0.987	0.991	0.045
Low environmental sensitivity group (*n* = 175)		206.104	49	0.091	0.965	0.974	0.035

**Table 5:** Hypotheses testing (H1 and H2; *n* = 408).

Hypothesis	*R* ^2^	*β*	SE
H1: Perceived restorativeness → Place identity	0.382	0.618^***^	0.075
H2: Perceived restorativeness → Place dependence	0.321	0.567^***^	0.071

^***^p < 0.001.

### 4.3. Invariance testing (H3)

The pooled sample was split around the median of the perceived restorativeness score (median = 17, max = 20, min = 8; total sum score of 4 environmental sensitivity items) to compare the differences between the high and low environmental sensitivity groups. If the perceived restorativeness score was the same or higher than the median, respondents were placed in the high environmental sensitivity group. If the perceived restorative-ness score was lower than the median, they were placed in the low environmental sensitivity group. As shown in Table 4, the models displayed a satisfactory fit for both the high environmental sensitivity group (*χ*^2^ = 126.967, df = 49, RMSEA = 0.061, NNFI = 0.987, CFI = 0.991, SRMR = 0.045) and the low environmental sensitivity group (*χ*^2^ = 206.104, df = 49, RMSEA = 0.091, NNFI = 0.965, CFI = 0.974, SRMR = 0.035).

**Figure 2:**
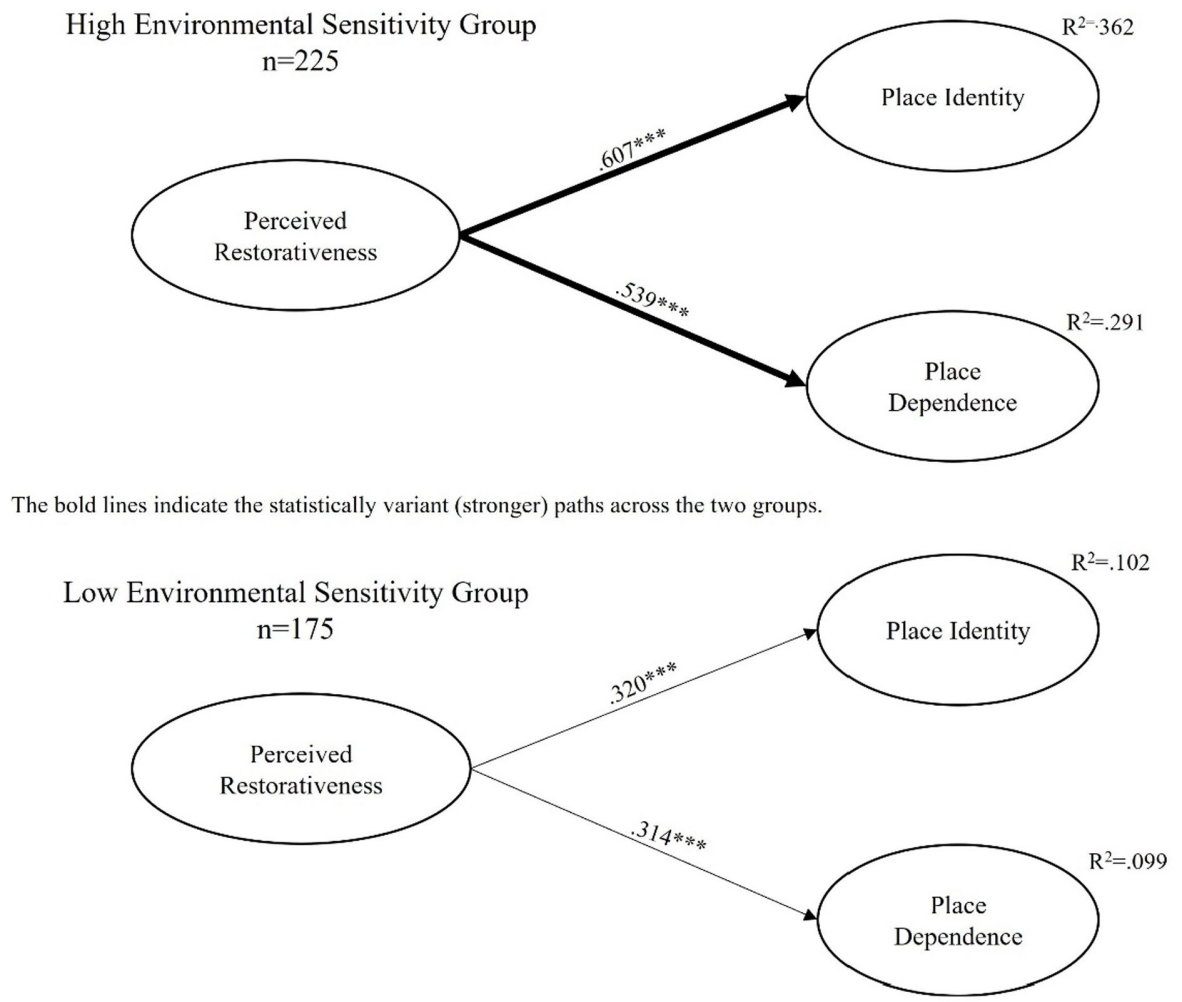
The results of invariance testing: moderating effect of environmental sensitivity on the relationship between perceived restorativeness and the two dimensions of place attachment.

To examine the moderating effect of perceived restorativeness and place attachment on the relationships tested in our measurement model, invariance testing was used to examine variation across the two groups (see Table 6 and Figure 2). The first step of invariance testing (measurement invariance) examined whether the factor structure of our hypothesized model was appropriate for the data. The model showed a good fit for the data (χ^2^ = 333.071, df = 98, RMSEA = 0.077, NNFI = 0.977, CFI = 0.981). Second, tests were conducted to examine whether the factor loadings were variant across the two groups (factor loading invariance). It was found that the five factor loadings were significantly different between the two subgroups (△χ^2^ = 43.322, *p* < 0.001, △df = 9). After allowing the free estimation of those loadings, the final model of the first stage was fixed (H2a: χ^2^ = 337.116, df = 102, RMSEA = 0.076, NNFI = 0.979, CFI = 0.975). Finally and most importantly, the invariance testing procedure examined whether the beta weights were significantly different between these groups (path coefficient invariance). Beta coefficients were first constrained to be invariant across the two groups (i.e., high vs. low environmental sensitivity) to analyze whether the imposition significantly affected the model fit. For the comparison of high and low environmental sensitivity groups, we found that imposing the invariance constraint significantly affected the model fit and the two paths were different (△χ^2^ = 51.375, *p* < 0.001, △df = 2). Therefore, there was a significant difference between high and low environmental sensitivity groups in terms of the regression coefficients. Regarding the relative strength of the paths from perceived restorativeness to place attachment dimensions (see Table 7), perceived restorativeness had a stronger positive influence on place identity (*β* = 0.607, *p* < 0.001) for the high environmental sensitivity group as compared to the low environmental sensitivity group (*β* = 0.320, *p* < 0.001). Also, perceived restorativeness influenced place dependence (β = 0.539, *p* < 0.001) more strongly for the high environmental sensitivity group as compared to the low environmental sensitivity group (*β* = 0.314, *p* < 0.001).

**Table 6:** Invariance tests (H3).

Invariance tests	*χ* ^2^	df	RMSEA	NNFI	CFI
(1) Measurement invariance	333.071	98	0.077	0.977	0.981
(2) Factor loading invariance	376.393	107	0.080	0.977	0.981
	△43.322^***^	△9			
2a final model^a^	337.116	102	0.076	0.979	0.975
(3) Path coefficient invariance	388.491	104	0.083	0.975	0.975
	△51.375^***^	△2			
3a final model^b^	333.116	102	0.076	0.979	983

^***^p < 0.001.

^a^A total 5 factors free estimation (RST2, RST3, RST4, PI3, PI4). ^b^A total 2 paths free estimation (*β*2 1, *β*3 1).

**Table 7:** Differences in path coefficients across two environmental sensitivity groups.

Path	Group	*β*	SE
Perceived restorativeness → Place identity	High environmental sensitivity group	0.607	0.191
	Low environmental sensitivity group	0.320	0.073
Perceived restorativeness → Place dependence	High environmental sensitivity group	0.539	0.216
	Low environmental sensitivity group	0.314	0.107

**Figure 3:**
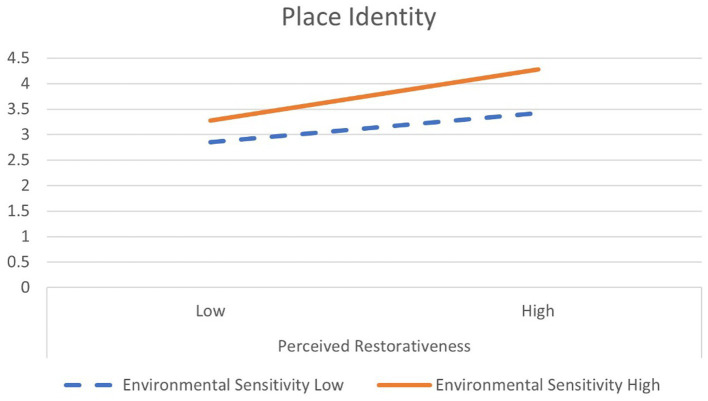
Moderation of environmental sensitivity: interaction plot for place identity.

Figure 3 is an interaction plot for the moderating effect of environmental sensitivity on the relationship between perceived restorativeness and place identity. In the case of place identity, when the perceived restorativeness was low, the average score was 2.85 for the group with low environmental sensitivity and 3.28 for the group with high environmental sensitivity. The average score difference was 0.43 for the groups with high-low environmental sensitivity. When the perceived restorativeness was high, the average of the group with low environmental sensitivity was 3.42, the average with high environmental sensitivity was 4.28, and the average score difference was 0.86. As such, the difference in the average score of place identity by a group of environmental sensitivity increased more than when the perceived restorativeness was high (0.86) compared to low (0.43). Therefore, the moderating effect of environmental sensitivity on place identity is more meaningful when perceived restorativeness is high. In addition, in the high and low perceived restorativeness, the high environmental sensitivity group was more effective in place identity than the low group.

**Figure 4:**
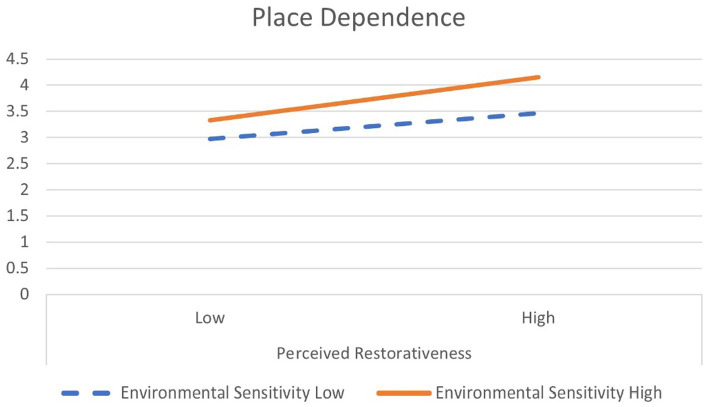
Moderation of environmental sensitivity: interaction plot for place dependence.

Concerning place dependence, when the perceived restorativeness was low, the average score was 2.97 for the group with low environmental sensitivity and 3.33 for the group with high environmental sensitivity (see Figure 4). The average score difference was 0.36 for the groups with high-low environmental sensitivity. When perceived restorativeness was high, the average of the group with low environmental sensitivity was 3.46, the average with high environmental sensitivity was 4.15, and the average score difference was 0.86. As such, the difference in the average score of place dependence by a group of environmental sensitivity increased more than when the perceived restorativeness was high (0.69) than low (0.36). Therefore, it can be interpreted that the moderating effect of environmental sensitivity on place dependence is more meaningful when the perceived restorativeness is high. In addition, in high and low perceived restorativeness, Environmental sensitivity in high groups is more effective in place dependence than in low groups.

## 5. Discussion

The present study examined how the perceived restorativeness of visitors to Jeju Gotjawal Provincial Park positively affected the two dimensions of place attachment (i.e., place identity and place dependence) and how the level of environmental sensitivity moderated the relationship between perceived restorativeness and place attachment. The results indicated that as visitors to Jeju Gotjawal Provincial Park felt restored, both place identity and place dependence increased. Furthermore, the higher the level of environmental sensitivity a visitor had, the stronger the association was of perceived restorativeness with place identity and place dependence. These results are discussed further in the following paragraphs.

First, the results indicated that, the perceived restorativeness of visitors to Jeju Gotjawal Provincial Park positively impacted their place identity. That means, as visitors experienced higher levels of perceived restorativeness, their place identity also increased. It was previously suggested that perceived restorativeness in a nature-based recreation setting can help increase place attachment (Ruiz et al., 2013; Ratcliffe and Korpela, 2016). In addition, people may develop attachment to a place where they had a lot of restorative experiences (Scopelliti and Giuliani, 2004). Korpela et al. (2009) also showed an association between attachment to place and psychological restoration. According to the study conducted by Devine-Wright and Howes (2010), it was found that the place attachment had a negative effect on the opposite of “naturalness.” They found a significant correlation between residents’ negative interpretations or evaluations of new industrialized facilities in the community and their actions against promoting such projects (e.g., signing petitions or writing letters). Furthermore, the study findings showed that bringing industrialized facilities such as wind farms into the community could threaten the place identity of their community. Place identity is one dimension of place attachment and includes both conscious and unconscious processes of confirming one’s identity within a favorite place or environment (Halpenny, 2010). The more a person likes a place, the stronger the feeling or thought that confirms part of one’s identity when he/she is placed in that environment. Increasing place identity means that the emotional bond that individuals share with the place increases, which can ultimately strengthen nature-friendly behavior (Halpenny, 2010; Scannell and Gifford, 2010a,b). For example, people who experience high place identity in Jeju Gotjawal Provincial Park naturally develop feelings that help them to cherish and love the place, which can lead to positive environmental behavior. While the association between perceived restorativeness and place attachment has been noted before by researchers, there have not yet been many studies that have examined the direct association between perceived restorativeness and one specific dimension of place attachment (e.g., place identity) An array of research indicates that natural experiences and ecotourism, particularly involving nature therapy and forest bathing, benefit human health (e.g., Mayfield, 2011; Bielinis et al., 2019; Farrow and Washburn, 2019; Furuyashiki et al., 2019). Thus, the effect of place identity on the perceived restorativeness of visitors to Jeju Gotjawal Provincial Park found in this study can contribute to further concretizing studies related to place identity in nature-based recreation areas.

Second, the results revealed that the higher the level of perceived restorativeness of visitors to Jeju Gotjawal Provincial Park, the higher their place dependence. Place dependence refers to cases where the reason for attachment is the functionality and usefulness of the place. Previous studies have shown that the higher the dependence of outdoor recreationists on a certain place, the stronger the idea that other similar leisure activity places cannot replace it (Kyle et al., 2005; White et al., 2008). Few studies have directly examined the relationship between perceived restorativeness and place dependence. However, there was a study that revealed that various values (e.g., aesthetic, wilderness, and spiritual value) of natural scenery are closely related to place dependence (Brown and Raymond, 2007). That study indicated that aesthetic, wilderness, and spiritual values positively predicted place dependence. Aesthetic value refers to the degree to which a person feels that the natural environment of the place he or she is visiting is a place that includes esthetically pleasing sights, sounds, and smells. In addition, wilderness value is the extent to which a person believes that a place is valuable because it has its own natural appearance. These two values (i.e., aesthetic and wilderness value) can be said to be closely associated with the perceived restorativeness felt by visitors to Jeju Gotjawal Provincial Park. When measuring perceived restorativeness in the present study, survey items such as “Jeju Gotjawal Provincial Park is a sufficiently attractive place” and “It is a place where you can explore nature” were used to measure aesthetic and wilderness values. This showed that perceived restorativeness, which is related to aesthetic and wilderness values in the previous study, can contribute to increased place dependence, which is the functional attachment to the park.

Third, the results indicated that among visitors to Jeju Gotjawal Provincial Park, the perceived restorativeness of those with high environmental sensitivity had a stronger effect on the two dimensions of place attachment than that of those with low sensitivity. This result implies that environmental sensitivity has a moderating effect on the relationship between perceived restorativeness and place attachment. Previous studies (Whitburn et al., 2019; Giusti and Samuelsson, 2020) indicated that people with higher positive environmental attitudes can have restorative experiences more frequently at an outdoor recreation setting. In addition, researchers (Budruk et al., 2009; Brehm et al., 2013) reported that the place attachment of people with positive environmental attitudes was stronger, thereby supporting the results of the present study. Whereas previous researchers have fragmentarily verified the relationship between environmental sensitivity and perceived restorativeness and the relationship between environmental sensitivity and place attachment, the present study empirically tested whether the relationship between perceived restorativeness and place attachment may become stronger or weaker depending on the level of environmental sensitivity. In other words, if visitors have higher environmental sensitivity, the influence of perceived restorativeness on place identity (feeling one’s identity strongly within a place) and place dependence (believing that a place effectively achieves one’s leisure purpose) can increase. Accordingly, it is necessary to actively provide visitors to the park with educational opportunities to develop sensitivity to the natural environment.

## 6. Implication and future research

The present study was conducted in Jeju Gotjawal Provincial Park, a park environment where visitors can experience perceived restorativeness and enjoyment while visiting environmentally significant recreation settings. Outdoor recreationists can learn about the preservation of natural resources by visiting the park. The present study explored whether visitors’ feelings of perceived restorativeness in the park could ultimately develop into feelings of attachment to the place. In addition, this study investigated whether the positive environmental attitude of visitors, that is, high environmental sensitivity, could further strengthen the relationship between perceived restorativeness and place attachment.

Planning a park management focusing on the perceived restorativeness emphasized by ART is necessary to efficiently allocate resources and maximize the benefits to visitors (Stack and Shultis, 2013). Physical health problems (e.g., chronic diseases) can be prevented in the long term by managing fatigue and stress from daily life (Swain, 2000), and the perceived restorativeness felt in nature can be a good remedy for health problems (Collado et al., 2017). One of the crucial determinants of mechanisms that boosts one’s physical and mental health is providing restorative and stress-reducing contact with nature (Orstad et al., 2020; Hazlehurst et al., 2022). At the public level, careful planning of park management and the provision of an optimal environment for visitors to experience perceived restorativeness can improve public health and reduce the financial burden of disease prevention and healthcare. Since scholars (Cohen et al., 2007) have already established that having accessible park areas and green spaces can be an important indicator of residents’ physical activity and stress levels, communities should find creative solutions to increase park area per capita. Scholars have also argued that if immediately increasing the park area per capita is impossible, events or activity programs in existing park areas should be diversified to help community residents make good use of the existing public resources. This point can be applied to the situation in South Korea as well. Since South Korea has a relatively small absolute land area, such programs should be developed so that many people can benefit from green spaces like Jeju Gotjawal Provincial Park. Previous research (Buckley and Cooper, 2022) also proposed considerable potential and profitable opportunities for the tourism sector to contribute to nature-based mental healthcare because an individual’s contact with nature can improve their mental health. Therefore, providing programs such as natural therapy to tourists visiting Jeju Gotjawal Provincial Park can be expected to improve their mental health. Furthermore, by providing more education programs, visitors can increase their environmental sensitivity, ultimately enhancing perceived restorativeness as well as attachment to the park environment. Since promoting environmental sensitivity has been proven to fosters healthy environmental citizenship (Priadi et al., 2018), it will be more effective to promote place attachment and environmental awareness by perceiving restorativeness in the park and promoting environmental appreciation simultaneously.

A limitation of the present study was that it only tested environmental sensitivity among various environmental attitudes. Studies hereafter can attempt to include other environmental attitudes, such as environmental knowledge and ecological worldview (Arcury et al., 1986; Du Plessis and Brandon, 2015). Furthermore, other place attachment dimensions such as emotional attachment and social bonding can be tested in addition to place dependence or place identity. Other factors, such as the duration of the visitor’s experience or personal relevance to the park, can also potentially influence the perceived restorativeness and place attachment. In addition, this study used the modified PRS (Yi, 2023), which was abbreviated and modified in Korea, instead of using the original PRS. However, since the original PRS measures a broader range of contents and includes more items, it will be necessary to actively reflect PRS in research on outdoor leisure activities in Korea. Lastly, future studies need to be expanded to visitors of various types of urban and community parks for further understanding of their restorative experiences and the development of place attachment in different settings.

## Data availability statement

The raw data supporting the conclusions of this article will be made available by the authors, without undue reservation.

## Ethics statement

The studies involving human participants were reviewed and approved by the Kyung Hee University Institutional Review Board (KHGIRB-21-400). The patients/participants provided their written informed consent to participate in this study.

## Author contributions

JY: conceptualization. JY and JJ: methodology, formal analysis, and writing—original draft preparation. JY, JJ, SL, and M-LK: writing—review and editing. All authors have read and agreed to the published version of the manuscript.

## Conflict of interest

The authors declare that the research was conducted in the absence of any commercial or financial relationships that could be construed as a potential conflict of interest.

## Publisher’s note

All claims expressed in this article are solely those of the authors and do not necessarily represent those of their affiliated organizations, or those of the publisher, the editors and the reviewers. Any product that may be evaluated in this article, or claim that may be made by its manufacturer, is not guaranteed or endorsed by the publisher.
